# The Late Mr. G. A. Cross

**Published:** 1904-11-05

**Authors:** 


### OBITUARY.

### THE LATE MR. G. A. CROSS, Secretary-Superintendent of the London Homœopathic Hospital.

WITH profound regret we announce the death of Mr. George Alfred Cross, the Secretary-Superintendent of the London Homœopathic Hospital, Great Ormond Street. Mr. Cross was attacked with syncope on October 15th while at the hospital, and had to be conveyed to his residence at Muswell Hill. His condition underwent material improvement, and it was expected that he would shortly be about again, but on Sunday, October 30, he had a relapse and died early the next morning.

Mr. G. A. Cross, who was 55 years of age, had been at the London Homœopathic Hospital for nearly 30 years, and throughout this long period (with the exception of the first two or three years) he held the chief executive position. In these 30 years the affairs of this hospital have undergone a complete transformation. The work of the institution was originally carried on in two or three converted dwelling-houses; now it is conducted in a building that for appointment and completeness will, perhaps, vie with any institution in the country. For this great progressive change the fullest credit is due to Mr. G. A. Cross. Moreover, he was possessed of mental attainments, administrative and organising capacity, of a high order, and these gifts he devoted to his hospital with such success that, in the opinion of those competent to judge, the institution was one of the best managed in the kingdom. Among his contemporaries in the hospital world, with whom he maintained most cordial relations, he was looked up to as a man of exceptional parts and held in the highest esteem. His death, which will come as a surprise to many, will be most acutely felt and will inflict a loss upon the Homœopathic Hospital which it will be difficult to fill.

